# Proteolytic Cleavage of Apolipoprotein E4 as the Keystone for the Heightened Risk Associated with Alzheimer’s Disease

**DOI:** 10.3390/ijms140714908

**Published:** 2013-07-17

**Authors:** Troy T. Rohn

**Affiliations:** Department of Biological Sciences, Science Building, Room 228, Boise State University, Boise, ID 83725, USA; E-Mail: trohn@boisestate.edu; Tel.: +1-208-426-2396; Fax: +1-208-426-4267

**Keywords:** apolipoprotein E, apoE4, Alzheimer’s disease, beta amyloid, neurofibrillary tangles, proteolysis, cleavage, neurodegeneration

## Abstract

Alzheimer’s disease (AD) is a progressive neurodegenerative disease characterized by microscopic lesions consisting of beta-amyloid plaques and neurofibrillary tangles (NFTs). The majority of cases are defined as sporadic and are likely caused by a combination of both genetic and environmental factors. Of the genetic risk factors identified, the 34 kDa protein, apolipoprotein (apo) E4, is of significant importance as APOE4 carriers account for 65%–80% of all AD cases. Although apoE4 plays a normal role in lipoprotein transport, how it contributes to AD pathogenesis is currently unknown. One potential mechanism by which apoE4 contributes to disease risk is its propensity to undergo proteolytic cleavage generating *N*- and *C*-terminal fragments. The purpose of this review will be to examine the mechanisms by which apoE4 contributes to AD pathogenesis focusing on the potential loss or gain of function that may occur following cleavage of the full-length protein. In this context, a discussion of whether targeting apoE4 therapeutically is a rationale approach to treating this disease will be assessed.

## 1. Introduction to Alzheimer’s Disease

According to the most recent facts and figures, Alzheimer’s disease (AD) affects approximately 5.2 million Americans, with the vast majority over the age of 65 [1]. Currently, AD is the sixth-leading cause of death in the United States and the fifth-leading cause of death for individuals age 65 and older [2]. AD is progressive neurodegenerative disorder characterized by an array of symptoms affecting memory and cognition. Some common symptoms of AD include memory loss that disrupts daily life, challenges in planning or solving problems, confusion with time or place, and changes in mood and personality [2]. Collectively these symptoms represent the term dementia, and AD is by far the most common cause of dementia in the United States accounting for 60 to 80 percent of cases [2]. In an effort to recognize the importance of early prevention and detection, new guidelines have been established for AD, the first since 1984 [3]. The three new stages of AD include: (1) preclinical AD that underscores the fact that the AD process begins before there are any overt symptoms of dementia; (2) mild cognitive impairment that includes patients with changes in memory and thinking ability but are not individuals classified as having AD. However, many of these patients will develop Alzheimer’s dementia; and (3) dementia, including mental impairments not as severe as those previously required for an Alzheimer’s diagnosis.

### 1.1. Pathology of Alzheimer’s Disease

**Figure 1:**
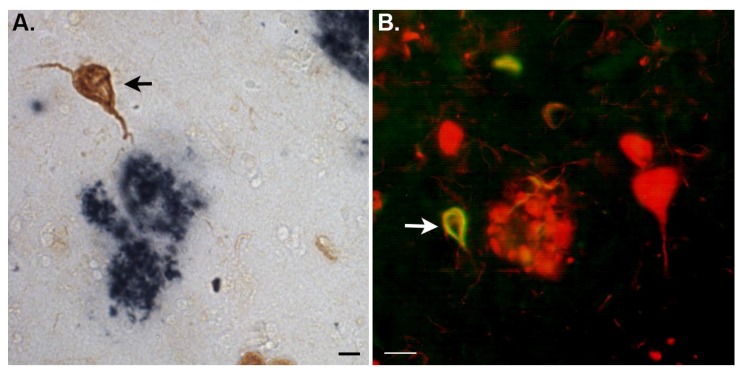
The amino terminal fragment of apolipoprotein (apo) E4 (apoE4) localizes to neurofibrillary tangles (NFTs) in the Alzheimer’s disease (AD) brain. (**A**) Representative bright field microscopy image from frontal cortex AD brain sections utilizing anti-Aβ antibody, clone 6E10 (blue-black) together with a custom synthesized antibody that specifically detects the amino terminal fragment of apoE4 (brown) revealed specific localization of this fragment within NFTs, in this case near an extracellular plaque labeled with an anti-Aβ antibody (arrow); (**B**) Double-label immunofluorescence using paired helical filaments (PHF-1) (red) as a marker for NFTs and the custom antibody to the amino terminal fragment of apoE4 (green) showed strong colocalization (yellow) in a subset of tangles in the AD brain (arrow). Scale bars are 10 μm for (**A**) and 20 μm for (**B**).

**Figure 2:**
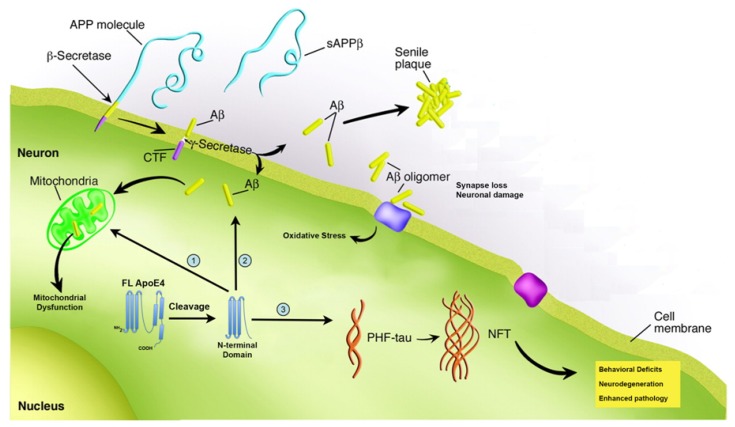
Proteolysis of apoE4 leads to a gain of function. The figure summarizes key features associated with AD including the formation of beta-amyloid by beta/gamma secretase and the deposition into extracellular plaques as well as formation of NFTs. A revision of the beta-amyloid hypothesis, as it has become known, is the role that oligomeric forms of beta-amyloid may play in disease progression. Thus, beta-amyloid oligomers now are thought to represent the cytotoxic species that can confer synapse loss, oxidative stress and neuronal damage. A key step in this process is mitochondrial disruption by beta-amyloid oligomers that may lead to the activation of apoptotic pathways in the AD brain. ApoE4 may promote the pathogenesis underlying AD following cleavage and generation of an *N*-terminal fragment. This fragment in turn may lead to a toxic gain of function of apoE4 in three ways: (1) Disruption of mitochondrial function by impairment of enzymes involved in the respiratory chain complex; (2) Promotion on the intracellular accumulation of beta-amyloid by stimulating cellular uptake; (3) induce tangle-like inclusions resembling neurofibrillary tangles. The cumulative end results of these processes may lead to enhanced pathology, neuronal deficits in memory and learning, and neurodegeneration. See main text for details.

Pathologically, AD is characterized by two microscopic lesions that include senile plaques and neurofibrillary tangles (NFTs). Both lesions must be present in order to have a post-mortem diagnosis of AD, and one never occurs without the other. Senile plaques are extracellular deposits of beta-amyloid, a 40–42 amino acid peptide that is derived following the processing of the amyloid precursor protein (APP) by beta and gamma secretases. Once formed, beta-amyloid has the propensity to self-aggregate into β-sheet structures that deposit extracellularly forming senile plaques (Figure 1). More recently, the beta-amyloid hypothesis has been revised to the “toxic beta-amyloid oligomer” hypothesis to reconcile the apparent lack of correlation between beta-amyloid in plaques and cognitive impairment [4]. This reformulation of the amyloid cascade hypothesis focuses on oligomeric aggregates of beta-amyloid as the prime toxic species causing AD in part because this form of beta-amyloid strongly correlates with the severity of dementia [5,6]. In addition, this oligomeric form of beta-amyloid is highly toxic and is the trigger for the loss of synapses and neuronal damage [7,8] (See Figure 2).

Specifically, beta-amyloid dimers have been isolated from the cortex of human AD cases and were the most abundant beta-amyloid species identified [9]. Importantly, Jin *et al.* demonstrated that natural dimers isolated from the AD brain induced phosphorylation of tau and neuritic degeneration that can be prevented following passive immunization with several beta-amyloid antibodies [9]. The next step in this process will be to identify the potential mechanisms by which beta-amyloid dimers induce neuritic changes within the AD brain. It has been hypothesized that beta-amyloid acting as dimers or multimers interface with neuronal cell membranes to induce tau modifications and subsequent neurodegeneration [10].

The other major pathological finding in AD is the presence of NFTs [11]. NFTs are primarily composed of aggregated phosphorylated tau protein and are a clinical feature not just in AD, but other diseases that are collectively referred to as “tauopathies” [12]. Tau normally functions to help maintain the stability of the cytoskeleton of neurons by binding to microtubules. However, upon hyperphosphorylation and posttranslational cleavage, tau loses its binding affinity for microtubules, leading to a destabilization of the cytoskeleton and self-assembly of tau into tangles of paired helical filaments (PHF-1) [13].

According to the beta-amyloid hypothesis, the accumulation and aggregation of beta-amyloid into toxic soluble oligomers is the first step leading to neuronal degeneration in AD [14]. Specifically, an important early molecular step is the lost of synapses, which correlates highly with the initial memory impairment observed in AD [15,16]. Intensive research over the last two decades has examined potential pathways activated by beta-amyloid aggregates that lead to synaptic dysfunction, NFT formation, and eventual cell death. Figure 2 summarizes some of the major findings on beta-amyloid-induced toxicity that begin with either beta-amyloid activation of apoptotic pathways or promotion of oxidative stress.

### 1.2. Risk Factors Associated with Alzheimer’s Disease

By far the greatest risk factor for AD is advancing age affecting 11 percent of those age 65 and older and 32 percent of people age 85 and older [1]. A small percentage of cases (<1%) are caused by known mutations in the *APP* gene or genes products involved in processing APP to form beta-amyloid and thus far, all identified mutations have the overall effect of enhancing the production of the beta-amyloid peptide (for a recent review, see [17]). The majority of these cases manifest before the age of 60 and therefore are classified as early-onset AD. Other known mutations that lead to early-onset AD include those to presenilin 1 (*PSEN1*) and presenilin 2 (*PSEN2*), whose gene products comprise the gamma-secretase complex [18,19]. The net effect of mutations to these two genes is enhanced production of beta-amyloid perhaps by shifting the cleavage site in APP [20].

Despite intensive research efforts, few candidate genes have been identified as potential risk factors for late-onset AD, representing those cases over the age of 65. A recent genetic analysis has suggested a link to mutations in various genes including *A2M* (encoding alpha-2-macroglobulin), *ABCA1* and *2* (encoding ATP-binding cassette transporters 1 and 2, respectively), *CLU* (encoding clusterin), *PICALM* (encoding the phosphatidylinositol binding clathrin assembly protein) and *SORL1* (encoding sortilin-related receptor gene) (for a review, see [21]).

Two other candidate genes that confer risk for late-onset AD include the *TREM2* and *APOE4* gene. Recently, two significant genetic variants of *TREM2* have been identified as increasing the risk for late-onset AD. The *TREM2* gene encodes the protein, triggering receptor expressed on myeloid cells 2 (TREM2). *TREM2* variants increase the risk for AD approximately 3-fold [22,23]. Activation of the TREM2 receptor protein on microglia has two important function consequences: (1) stimulation of phagocytosis activity and (2) decreased microglial proinflammatory responses [24]. Collectively, the TREM2 protein may function to help aid microglia to clear damaged or apoptotic cells, cellular debris and help resolve damage-induced inflammation. It has been suggested that both of these *TREM2* variants may lead to the expression of a non-functional TREM2 protein and loss of these critical functions in microglia. The other major risk factor for developing late-onset AD is harboring the apolipoprotein E4 (APOE4) allele [25]. Although harboring the *APOE4* allele clearly enhances the risk for AD, the mechanism by which the apoE4 protein contributes to AD pathogenesis is not known. The focus of this review is to highlight some of the putative mechanisms by which apoE4 contributes to AD pathogenesis.

## 2. Apolipoprotein E: Structure and Function

Human apoE is polymorphic with three major isoforms, apoE2, apoE3, and apoE4, which differ by single amino acid substitutions involving cysteine-arginine replacements at positions 112 and 158 [26]. Harboring the *APOE3* allele is believed to neither increase nor decrease one’s risk of AD, while having the *E2* form may decrease one’s risk. In contrast, inheritance of one copy of the *APOE4* allele increases disease risk fourfold, while two copies raises the risk tenfold [25]. It is noteworthy that 65%–80% of all AD patients have at least one *APOE4* allele [27,28]. Structurally, apoE4 is a 34 kDa protein composed of 299 amino acids and contains two major domains, referred to as the *N*-terminal (~20 kDa) and *C*-terminal (~10 kDa) domains, which are connected by a short hinge region [29] (Figure 2). Functionally, in the CNS, apoE is produced by a variety of cells including astrocytes, and transports cholesterol to neurons via apoE receptors, which are members of the low-density lipoprotein (LDL) receptor family [30,31]. It has been proposed that because apoE is the major cholesterol transporter in the brain (see below) and therefore is essential for synaptogenesis in neurons, then apoE-isoform-dependent differences in these processes may negatively impact synaptic plasticity or recovery of neurons from neurodegeneration as might occur in AD [32,33].

## 3. Proteolysis of apoE4 as a Mechanism Underlying Pathogenesis in AD

Although apoE4 plays a normal role in lipoprotein transport, how it contributes to AD pathogenesis is currently unknown. An emerging clue to this puzzle are data demonstrating that apoE4 is highly susceptible to proteolysis compared to the other major isoforms of apoE [34]. In this regard, the hinge region of apoE4 has multiple protease-sensitive sites and although the exact nature of the protease involved in cleaving apoE4 is unknown several candidates have been reported including cathepsin D [35], a chymotrypsin-like protease [34], and aspartic proteases [36]. Although evidence suggests the hinge region of apoE4 represents a region of proteolytic susceptibility, it is clear based on the number of different fragments demonstrated to occur in human brain homogenates, that other regions within apoE4 including the *N*- and *C*-terminal domains are also susceptible to cleavage [37]. In mouse models of AD, proteolytic cleavage of apoE4 has been demonstrated in neurons, not astrocytes, to accumulate in an age-dependent manner, and cause AD-like memory deficits [34,38,39]. These data support the hypothesis that intraneuronal proteolytic cleavage of apoE4 could promote the neuropathology and neurodegeneration in AD brains. Supporting a role of proteolytic cleavage of apoE4 are studies demonstrating the presence of apoE4 fragments (14–20 kDa) in the AD brain [34,40–42].

Intriguing, is following the cleavage of apoE4 into two distinct domains, each domain appears to localize to specific pathological lesions in the AD brain. Thus, the *C*-terminal domain of apoE4 has been implicated in binding to beta-amyloid and is localized to plaques [36,40,42]. On the other hand, the *N*-terminal domain preferentially localizes within NFTs [34]. We recently confirmed the specific localization of the *N*-terminal domain of apoE4 to NFTs in the AD brain by synthesizing a site-directed antibody to a putative cleavage site within apoE4 at position D172. This antibody, which detects only the *N*-terminal fragment of apoE4 localized exclusively within NFTs of the AD brain [42] (Figure 1). The high susceptibility of apoE4 to proteolytic cleavage may be the driving force behind the enhanced risk for AD in individuals who are either heterozygous or homozygous for the *APOE4* allele. Emerging data suggests the cleavage of apoE4 may increase AD risk in two ways, either through a loss of function or gain of toxicity.

### 3.1. Proteolysis of apoE4 Leads to Loss of Function

Variations in the *APOE* gene sequence results in three common alleles, *E2*, *E3*, and *E4*, represented with frequencies of 8%, 77%, and 15%, respectively, in the population [43]. As stated above, apoE4 is highly susceptible to proteolysis as compared to the other isoforms and this cleavage may result in loss of function. An important role of apoE is its ability to transport cholesterol within the CNS. CNS apoE is synthesized locally in the brain by neurons [44], astrocytes [45], and microglia [46]. With respect to neurons, Xu *et al.* eloquently demonstrated in green fluorescent apoE protein reporter mice that under normal conditions neurons express very little apoE protein. However, in response to excitotoxic injury, neurons responded with a significant increase in apoE expression [47]. Importantly, the plasma pool of apoE does not appear to exchange with the brain pool due to the presence of the blood brain barrier and consequently, there is no mixing of cholesterol pools between the brain and the periphery [48]. Transport of cholesterol by apoE may provide neurons the necessary cholesterol required for synapse formation, plasticity, and repair [49]. Moreover, synaptic development and plasticity are governed by the availability of cholesterol and decline with aging, suggesting a pivotal role for apoE in aging [50].

In addition to cholesterol, current evidence suggests that apoE plays an important role in transporting beta-amyloid in the AD brain. It has been suggested that apoE binds beta-amyloid directly to influence its clearance (for review, see [51]). Specifically, it has been proposed that astrocyte-derived apoE is critical for the degradation and clearance of deposited beta-amyloid, and that this process may be impaired in AD [52]. Recently, Bien-Ly *et al.* crossed an AD mouse model with mice that express *C*-terminal-truncated apoE4 and showed these mice had a lower affinity for beta-amyloid and a reduced ability to clear beta-amyloid [39]. In addition, the *C*-terminal-truncated fragment of apoE4 acted in concert with beta-amyloid to promote behavior deficits in these mice [39]. Further support for a critical role of apoE-mediated removal of beta-amyloid are recent studies demonstrating the administration of anti-cancer drug, bexarotene in an animal model of AD, enhanced clearance of beta-amyloid and reversed cognitive impairments in an apoE-dependent manner [53,54]. However, the exact mechanism by which apoE enhances clearance of beta-amyloid plaque loads is unclear [55–57]. It has been proposed that apoE can bind to beta-amyloid and undergo endocytosis via the astrocyte low-density lipoprotein receptor-related protein (LRP) [58,59].

**Figure 3:**
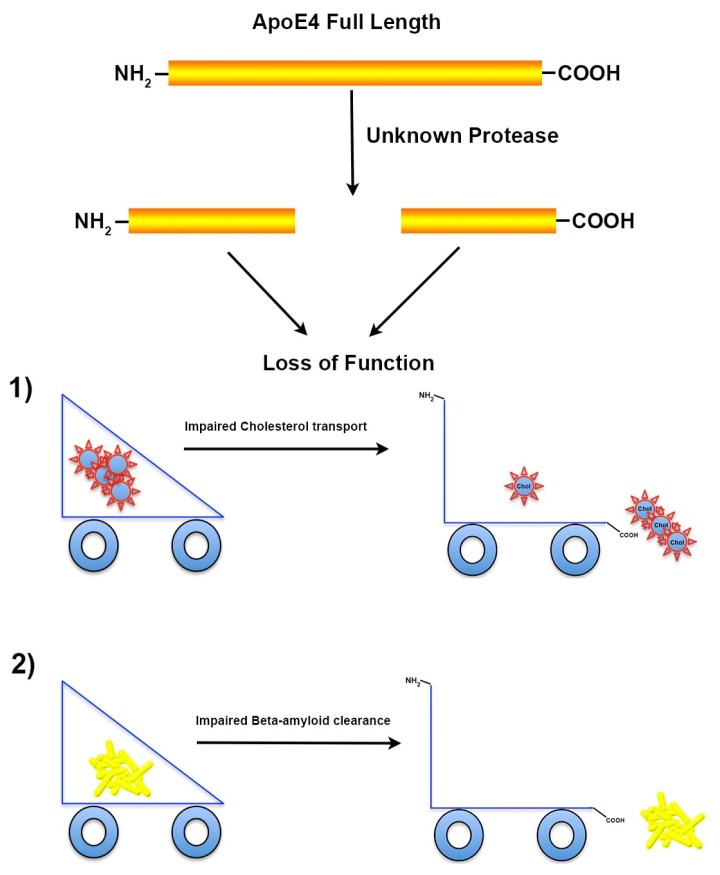
Proteolysis of apoE4 leads to a loss of normal function. Following cleavage of apoE4 by as yet an unidentified protease, could hypothetically lead to a loss of function of apoE4; including: (1) Impairment of cholesterol transport. In the CNS, apoE is one of the major lipid acceptors and functions to shuttle cholesterol to and from cells to generate high-density lipoprotein (HDL) particles. Loss of this function could deplete HDL-cholesterol that is essential for synaptogenesis and neurite outgrowth in neurons and thereby limit the recovery from neurodegeneration observed in AD; (2) AD is associated with impaired clearance of beta-amyloid from the brain, a process that is normally facilitated by apoE. In this regard, apoE4 is known to bind directly to oligomeric beta-amyloid enhancing its clearance out of the CNS and although it does this very poorly as compared to apoE2 and apoE3, proteolytic cleavage could further exacerbate this important role in individuals harboring the apoE4 allele in AD.

Given the preponderance of data suggesting a critical role of apoE in enhancing beta-amyloid clearance, cleavage of apoE4 following its expression may cause a loss of this important function leading to accumulation and aggregation of toxic beta-amyloid species. Indeed, a recent study has demonstrated a differential regulation of beta-amyloid clearance by isoforms of apoE, with apoE3 being more efficient in this process than apoE4 [60]. Taken together, the expression apoE4, susceptibility of cleavage and eventual loss of function both in cholesterol transport and beta-amyloid clearance may explain the heightened risk for developing AD associating with expression of the *APOE4* allele (Figure 3).

### 3.2. Proteolysis of apoE4 Leads to a Toxicity Gain of Function

Proteolytic cleavage of apoE4 may not only lead to loss of function, but current evidence suggests that generated fragments can produce a toxic-gain of function. For example, cleavage of apoE4 produces *N*- and *C*-terminal fragments that are neurotoxic in nature [40,61–63]. Tolar *et al.* showed that not only is a 22 kDa *N*-terminal fragment of apoE4 neurotoxic, but that it is significantly more toxic than the same fragment derived from the E3 isoform [62]. It has been postulated that the source of these toxic fragments occurs following intraneuronal processing of apoE4 [40]. Not only have these fragments been shown to be neurotoxic, but also may actually promote the underlying pathology associated with AD. Thus, *N*-terminal fragments of apoE4 can facilitate the production of tangle-like inclusions, reminiscent of early NFTs both *in vitro* and in animal models [34,61,63]. Similarly to the neurotoxic actions, it is neuron-specific proteolytic cleavage of apoE4 that is associated with increased phosphorylation of tau and the formation of tangle-like inclusions [38]. In addition to promoting the formation of tau filaments, apoE4 fragments have been shown to promote intracellular accumulation of the beta-amyloid peptide (1–42) [64]. In this regard, the authors found that a specific fragment of apoE4 promotes the cellular uptake of extracellular beta-amyloid, *in vitro*, and this led to the formation of reactive oxygen species (ROS) [64]. It is noteworthy that these *in vitro* findings support post-mortem observations in human AD brain tissue indicating an accumulation of intraneuronal beta-amyloid, which was correlated highly with the *APOE4* genotype [65].

Besides the propensity of apoE4 fragments to promote the pathology associated with AD, there have been several reports of that apoE4 may lead to mitochondrial dysfunction [61,66,67]. Specifically, Nakamura *et al.* have shown that a specific *N*-terminal fragment of apoE4 is capable of binding mitochondrial proteins associated with oxidative phosphorylation [67]. This fragment bound to ubiquinol cytochrome c reductase core protein 2, cytochrome C1, and cytochrome c oxidase subunit 4 isoform 1 leading to altered enzymatic activities as well as overall mitochondrial dysfunction [67]. These findings on mitochondria function have been supported by a recent study using postmortem brain tissue, whereby reduced mitochondrial activity was observed in young adult carriers of the *APOE4* allele [68].

Functionally, apoE4 fragments have been shown to cause learning and memory deficits in transgenic mice. For example, apoE4 knock-in mice displayed an age-dependent decrease in GABAergic neurons, which was mediated through the GABA_A_ receptor [63]. The loss of these neurons resulted in learning and memory deficits in these mice that were rescued following treatment of mice with the GABA_A_ receptor antagonist, picrotoxin [63]. Harris *et al.* demonstrated that expression of a *C*-terminal-truncated apoE4 fragment in mice resulted in behavioral deficits as well as neurodegeneration [34]. Finally, Bien-Ly *et al.* showed that expression of a *C*-terminal-truncated fragment of apoE4 not only resulted in behavior deficits in transgenic AD mice, but that this fragment acts in concert with beta-amyloid to elicit this effect [39]. In addition, expression of this fragment in AD mice led to a failure to properly clear beta-amyloid resulting in an increased beta-amyloid deposition [39]. Taken together these results provide strong support for a toxic-gain of function for cleaved fragments of apoE4 both in terms of enhancing the pathological mechanisms underlying AD, but also leading to functional declines in learning and memory. Figure 2 summarizes the toxic-gain of action of apoE4 following proteolytic cleavage in the hinge region of the protein.

## 4. Targeting apoE4 Therapeutically for the Treatment of Alzheimer’s and Concluding Remarks

The *APOE4* allele represents the greatest genetic risk factor for the development of late-onset AD. Although clearly a risk factor, how apoE4 contributes to disease pathogenesis is currently not known. The purpose of this review was to assess the current state of apoE4 with regards to studies that provide a mechanistic foundation for how expression of the *APOE4* allele confers Alzheimer’s susceptibility. In this regard, emerging evidence strongly suggests the link between apoE4 and disease risk is proteolysis of apoE4 generating *N*- and *C*-terminal fragments that may lead to loss of normal function or a toxic-gain of function with each possibility not mutually exclusive of each other. In either case, therapeutics designed to either limit proteolysis or replace cleaved apoE4 that is no longer effective may be a suitable treatment option. Recently, it has been shown that bexarotene (Targretin) enhances the expression of apoE, promotes the clearance of beta-amyloid, and reverses the behavioral deficits associated with AD Tg2576 transgenic mice [53]. Bexarotene is already approved by the USA Food and Drug Administration as an anti-cancer agent and has a favorable safety profile [69]. This is an important consideration as drugs that are already approved have a much clearer track to be tested in clinical trials. What is the putative mechanism by which bexarotene mediates the clearance of beta-amyloid? The authors propose that bexarotene induces the expression of the apoE through an action on retinoid X receptors, which act as transcription factors. The enhanced expression of apoE in turn, facilitates and enhances normal beta-amyloid clearance mechanisms [53].

Several questions remain, however, on this proposed action of bexarotene. First, what remains unclear is whether bexarotene increases the expression of all isoforms of apoE because treatment of individuals who express at least one apoE4 allele may not respond favorably, especially if a toxic-gain of function is the mechanism underlying the enhanced risk of AD. Second, the study by Cramer *et al.* has recently been challenged by several groups who have been unable to recapitulate central aspects of the study [54,56,70]. These conflicting reports suggest more studies are warranted with regards to bexarotene and its potential efficacy as treatment option for AD patients.

An alternative treatment approach first suggested by the group at the Gladstone Institute of Neurological Disease (San Francisco, CA, USA) is the development of small-molecule structure correctors that could be employed to prevent the cleavage of apoE4. The basis for this approach is that it has been suggested apoE4’s detrimental effects result from altered protein conformation, making it more likely to be cleaved and producing neurotoxic fragments [71]. Recently, this group has identified several such compounds that reverse the neurotoxic actions of apoE4, *in vitro* [72,73]. The efficacy of these compounds appears to be directly correlated with their ability to bind apoE4 and essentially keep it in an “apoE3” form that is unable to be proteolyzed [71]. The Gladstone group has also published tantalizing *in vivo* data showing these apoE4 structure correctors are not toxic and result in a 20%–25% decrease in the levels of apoE4 fragments in the brain of apoE4-expressing mice [71]. Their data suggest that structure-corrector-based therapies targeted towards apoE4 carriers could provide a therapeutic avenue for the treatment of AD. Perhaps the time has come to flesh out these potential treatment opportunities considering that 65%–80% of all AD patients have at least one apoE4 allele [28] and apoE4 expression decreases the age of onset, resulting in cognitive deficits 7–8 years earlier in individuals carrying one allele and 15–16 years earlier in those carrying two alleles [74].
